# Correlation between mutational status and survival and second cancer risk assessment in patients with gastrointestinal stromal tumors: a population-based study

**DOI:** 10.1186/s12957-015-0474-0

**Published:** 2015-02-13

**Authors:** Jordi Rubió-Casadevall, Joan Lluis Borràs, Maria Carme Carmona-García, Alberto Ameijide, Allan Gonzalez-Vidal, Maria Rosa Ortiz, Ramon Bosch, Francesc Riu, David Parada, Esther Martí, Josefina Miró, Juan Jose Sirvent, Jaume Galceran, Rafael Marcos-Gragera

**Affiliations:** Medical Oncology Department, Institut Català d’Oncologia de Girona, Girona, Spain; Descriptive Epidemiology, Genetics and Cancer Prevention Group, Biomedical Research Institute (IDIBGI), Girona, Spain; Faculty of Medicine, University of Girona (UdG), Girona, Catalonia Spain; Medical Oncology Department, Hospital Universitari Sant Joan, Institut d’Investigació Sanitària Pere Virgili (IISPV), Universitat Rovira i Virgili, Reus, Spain; Tarragona Cancer Registry, Fundació per a la Investigació i Prevenció del Càncer (FUNCA), IISPV, Universitat Rovira i Virgili, Tarragona, Spain; Faculty of Medicine, Universitat Rovira i Virgili, Tarragona, Spain; Red Temática de Investigación Cooperativa en Cáncer (RTICC), Girona, Spain; Epidemiology Unit and Girona Cancer Registry (UERC), Oncology Coordination Plan Department of Health Government of Catalonia, Girona, Spain; Department of Pathology, Hospital de la Santa Creu i Sant Pau, Autonomous University of Barcelona, Barcelona, Spain; Department of Pathology, University Hospital Josep Trueta, Girona, Spain; Department of Pathology, University Hospital Verge de la Cinta, Institut d’Investigació Sanitària Pere Virgili (IISPV), Fundació Dr. Ferran (FF), Tortosa, Spain; Department of Pathology, University Hospital Sant Joan, Institut d’Investigació Sanitària Pere Virgili (IISPV), Reus, Spain; Department of Pathology, Hospital Santa Tecla, Tarragona, Spain; Department of Pathology, Clinica Girona, Girona, Spain; Department of Pathology, University Hospital Joan XXIII, Tarragona, Spain

**Keywords:** Gastrointestinal stromal tumors, Sarcoma, Survival, Epidemiology, Registries, C-KIT, BRAF, PDGFR, Second primary cancer

## Abstract

**Background:**

Gastrointestinal stromal tumors are sarcomas of the digestive tract characterized by mutations mainly located in the c-KIT or in the platelet-derived growth factor receptor (PDGFR)-alpha genes. Mutations in the BRAF gene have also been described. Our purpose is to define the distribution of c-KIT, PDGFR and BRAF mutations in a population-based cohort of gastrointestinal stromal tumors (GIST) patients and correlate them with anatomical site, risk classification and survival. In addition, as most of the GIST patients have a long survival, second cancers are frequently diagnosed in them. We performed a second primary cancer risk assessment.

**Methods:**

Our analysis was based on data from Tarragona and Girona Cancer Registries. We identified all GIST diagnosed from 1996 to 2006 and performed a mutational analysis of those in which paraffin-embedded tissue was obtained. Observed (OS) and relative survival (RS) were calculated according to risk classifications and mutational status. Multivariate analysis of variables for observed survival and was also done.

**Results:**

A total of 132 GIST cases were found and we analyzed mutations in 108 cases. We obtained 53.7% of mutations in exon 11 and 7.4% in exon 9 of c-KIT gene; 12% in exon 18 and 1.9% in exon 12 of PDGFR gene and 25% of cases were wild type GIST. Patients with mutations in exon 11 of the c-KIT gene had a 5-year OS and RS of 59.6% and 66.3%, respectively. Patients with mutations in exon 18 of the PDGFR gene had a 5-year OS and RS of 84.6% and 89.7%. In multivariate analysis, only age and risk group achieved statistical significance for observed survival. GIST patients had an increased risk of second cancer with a hazard ratio of 2.47.

**Conclusions:**

This population-based study shows a spectrum of mutations in the c-KIT and PDGFR genes in GIST patients similar to that previously published. The OS and RS of GIST with the exon 18 PDGFR gene mutation could indicate that this subgroup of patients may be less aggressive and have a good prognosis, although less sensitive to treatment at recurrence. In our study, GIST patients have an increased risk of developing a second neoplasm.

## Background

Gastrointestinal stromal tumors (GIST) are the most common sarcomas of the digestive tract, with an incidence of 1.1 to 1.4 cases/100,000 inhabitants/year [1-3]. It was initially described by Mazur and Clark [4] in 1983 and develops from the interstitial cells of Cajal in the digestive tract. In 1998, Hirota and colleagues [5] identified a gain-of-function mutation in the c-KIT gene that characterized this tumor. Mutations in the c-KIT gene led to constitutive activation of a tyrosine kinase receptor that induced oncogenesis [6]. In some GIST in which mutations in c-KIT have not been identified, oncogenic mutations have been reported in the gene encoding the platelet-derived growth factor receptor-alpha (PDGFR-α) [7].

Mutations in the c-KIT and PDGFR-α genes are clustered in just a few exons. The type of mutation has been correlated with prognosis, localization of primary tumors, initial sensitivity and later resistance to imatinib treatment and association with hereditary syndromes [8,9].

BRAF gene mutations had initially been described in 13% of wild-type GIST [10], although recently they have also been reported in 2% of GIST carrying activating KIT and PDGFR-α mutations and conferring a worse prognosis and resistance to imatinib treatment [11]. Some other mutations in oncogenes such as PTEN, KRAS and NTRK2 have been described, especially in wild-type KIT GIST [12].

In 2001, the characteristics of GIST and the diagnostic approach were established by expert consensus [13]. Since this meeting, a positivity in c-KIT immunohistochemistry test has been mandatory for diagnosis of GIST, although some cases, about 5%, may be c-KIT negative. Also, GIST patients were classified into different groups regarding risk of malignant behavior according to tumor size and number of mitoses, with different survival for each [1].

The introduction of imatinib mesilate in 2002 produced a remarkable change in the results of therapy with GIST patients [14].

Focusing on the epidemiology of this type of neoplasm, our purpose is to define the distribution of KIT, PDGFR-alpha and BRAF mutations in a population-based cohort of GIST and correlate them with risk classifications and survival.

GIST patients are frequently diagnosed of a second cancer probably due to the long survival of the less aggressive tumors. In addition, gastric GIST has been described as Carney triad disease including also extra-adrenal paraganglioma and pulmonary chondroma and an association between GIST and type 1 neurofibromatosis has also been established. Those inherited GIST are in most cases not related to c-KIT or PDGFRA mutations [15,16]. We also present our results in a population-based evaluation of risk of secondary malignances after GIST.

## Methods

Our analysis was based on data from two cancer registries in the North-East of Spain. The Girona Cancer Registry (GCR) is a population-based cancer registry covering the province of Girona, which began its activity in 1994. In this area we analyzed the time period 1996 to 2006. The Tarragona Cancer Registry (TCR) is a population-based cancer registry that has registered all malignant tumors in the province of Tarragona since 1980. In this area we analyzed the time period 1998 to 2006. These cancer registries record cancers according to International Agency for Research on Cancer (IARC) guidelines [17].

We decided to analyze both areas together because they are geographically close and their population has very similar demographic characteristics. According to census data from the Catalan Institute of Statistics (available at URL: www.idescat.cat), in 2006 Tarragona had 729,671 inhabitants (50.6% male) and the age distribution was 14.3% in the range 0 to 14 years old, 68.0% in the range 15 to 64 years-old and 17.8% over 64 years old. At the same time, Girona had 681,387 inhabitants (50.5% male) and the age distribution was 14.4% in the range 0 to 14 years old; 67.7% in the range 15 to 64 years old and 17.9% over 64 years old.

As some of low-risk GIST tumors had been considered benign in the past and could have been missed by Cancer Registries [2], we additionally requested that the pathology departments of both areas reviewed all cases of sarcoma of the intestinal tract and reclassified them performing immunohistochemistry using CD117, CD34 and other markers such as desmin, S-100 protein and smooth muscle actin to correctly diagnose GIST.

Clinical data of patients was obtained and GIST were classified by risk group of malignant behavior as defined in the National Institutes of Health GIST workshop in April 2001 and published by Fletcher et al. [13] in 2002, which was the classification system widely used in the years covered by our analysis. We also show data using the Armed Forces Institute of Pathology (AFIP) classification defined by Miettinen et al. [18], for which we reclassified all cases.

We asked for paraffin-embedded tissue samples of all cases and searched mutations in exons 9, 11, 13 and 17 in the KIT gene and exons 12 and 18 in the PDGFR-α gene. In wild-type cases for both genes, we searched for BRAF gene mutations.

Formalin-fixed, paraffin-embedded tumor blocks were reviewed by expert pathologists for quality and tumor content and a single representative tumor block from each patient, containing at least 80% of neoplastic cells, was selected. Genomic DNA was extracted using the QIAamp DNA FFPE Tissue Kit (Qiagen, Hilden, Germany) as specified by the manufacturer’s instructions.

KIT mutations in exons 9, 11, 13 and 17, PDGFRα mutations in exons 12 and 18 and BRAF mutations in exon 15 were assessed on tumor DNA. Mutational analysis was performed by polymerase chain reaction (PCR) amplification and subsequent sequencing analysis. Mutational analysis was performed using previously reported PCR conditions and primers (Table 1) for exons 9, 11, 13 and 17 of the KIT gene [19,20], for exons 12 and 18 of the PDGFRα gene [21,22] and for exon 15 of the BRAF gene [10]. Each sample was capillary-electrophoresed on an ABI PRISM 310 Genetic Analyzer (Applied Biosystems, Foster City, USA).

**Table 1:** **Primer sequences used for PCR**

**Location**	**Primer sequence**	**Size (in bp)**
*c-KIT* exon 9	F: 5′-TCCTAGAGTAAGCCAGGGCTT-3′	283
	R: 5′-TGGTAGACAGAGCCTAAACATCC-3′	
*c-KIT* exon 11	F: 5′-CCAGAGTGCTCTAATGACTG-3′	215
	R: 5′-AGCCCCTGTTTCATACTGAC-3′	
*c-KIT* exon 13	F: 5′-GCTTGACATCAGTTTGCCAG-3′	193
	R: 5′-AAAGGCAGCTTGGACACGGCTTTA-3′	
*c-KIT* exon 17	F: 5′-GGTTTTCTTTTCTCCTCCAACC-3′	203
	R: 5′-GATTTACATTATGAAAGTCACAGG-3′	
*PDGFRA* exon 12	F: 5′-CCAGTTACCTGTCCTGGTCAT-3′	183
	R: 5′-GGAGGTTACCCCATGGAACT-3′	
*PDGFRA* exon 18	F: 5′-TACAGATGGCTTGATCCTGAGT-3′	212
	R: 5′-AGTGAAGGAGGATGAGCCTG-3′	
*BRAF* exon 15	F: 5′AAACTCTTCATAATGCTTGCTCTG 3′	200
	R: 5′GGCCAAAAA TTTAATCAGTGG A 3′	

bp, base pair.

Follow-up time was calculated as the time between the date of diagnosis and the date of death, if the patient was dead, or data from the last check-up if the patient was alive. We also obtained patients’ vital status by means of record linkage to the Mortality Registry of Catalonia and the Spanish National Death Index, updated until 31 December 2012.

Relative survival (RS) was defined as the ratio between observed survival (OS) and expected survival and was calculated to express the probability of cancer patients surviving after adjustment for competing causes of death [23]. Survival was estimated using only first tumors. RS rates were calculated using the Hakulinen method [24]. Expected survival was estimated using mortality tables for Catalonia.

The chi-square test was used to determine whether the type of mutation was related to the tumor site, Fletcher risk group and AFIP risk group.

Using Cox regression models, age, sex, type of mutation, risk group according to Fletcher’s or AFIP’s classification and localization were investigated as potential prognostic factors for impact on observed survival in multivariate analyses. The same variables were analyzed in univariate model using log-Rank test.

In terms of evaluating the risk of developing a second cancer after a diagnosis of GIST, we performed a retrospective analysis in both cohorts. The IARC-IACR recommendations for the definition of multiple primaries were used as criteria for inclusion or exclusion of tumors as second primary cancers [25].

The standardized incidence ratio (SIR) was calculated as the ratio between the observed number of second cancers and the number that would be expected if patients in the cohort experienced the same cancer incidence rates as the general reference population. The observed number of cases included all second cancers diagnosed in the cohort patients, excluding non-melanoma skin cancers. If the second cancer was a non-melanoma skin cancer, the third cancer was considered as the ‘second’. Multiple cancers diagnosed before GIST were excluded. The expected number of cases was computed multiplying the cumulated person-years observed by the incidence rates by cancer site, 5-year age group and calendar-year of the general population of Tarragona and Girona. Person-years observed (PYO) at risk were defined as the period going from the first cancer diagnosis to the first date among the following: the date of second cancer diagnosis, the date of death or the date of last known vital status (31 December 2012). The assumption that the observed number of subsequent cancers followed a Poisson distribution was used to calculate 95% confidence intervals.

All the analysis was computed using R software.

This study was approved by the Ethic Committee of each referral Hospital of the whole area (Hospital Sant Joan in Reus, Hospital Joan XXIII in Tarragona and Hospital Josep Trueta in Girona).

## Results

We identified 75 GIST incident cases in the Girona Cancer Registry for the period 1996 to 2006. The crude rate of incidence in this area and period was 1.17 cases/100,000 inhabitants/year (World age-standardized rate of 0.68 cases and European age-standardized rate of 0.93).

In the Tarragona Cancer Registry, 57 GIST incident cases were identified for the period 1998 to 2006, corresponding to a crude rate of incidence of 1.01 cases/100,000 inhabitants/year (World age-standardized rate of 0.62 and European age-standardized rate of 0.85).

### Cohort characteristics

A total of 132 GIST cases were found for the two populations. Of these, 67 were in men (55.8%) and 53 in women (44.2%). Median age at diagnosis was 64.5 years old.

Seventy-six cases (57.6%) were localized in the stomach, 47 (35.5%) in the small intestine, 6 (4.5%) in the peritoneum, two in the rectum (1.5%) and one in the esophagus (0.8%).

Forty cases (30.3%) were classified as low or very low risk of malignant behavior according to the classification proposed by Fletcher et al. [13], 28 cases (21.2%) were of intermediate risk, 46 cases (34.8%) were of high risk, 14 cases (10.7%) were diagnosed with metastatic disease and in 4 cases (3%) we did not have enough information to classify.

Twenty-three of the 132 patients of the cohort (17.4%) had been treated with imatinib at the end of study follow-up. In six patients, treatment was initiated upon diagnosis of metastatic disease. In seventeen patients, it was initiated at recurrence following initial surgery. Of these, 14 were initially classified as high risk of malignant behavior GIST, one as intermediate risk and two as low risk. Any patient was treated in adjuvant setting. As some of the patients died before the widespread use of imatinib in GIST, the impact of treatment on survival could not be evaluated in our series.

The median follow up time was 97 months (8.09 years).

### Mutational analysis

In GCR, we obtained paraffin-embedded tissue of 65 cases, corresponding to 86.6% of the total incidence. All tissues corresponded to the primary tumor and were obtained at the time of diagnosis or first surgical treatment. In two paraffin-embedded tissues, there was insufficient DNA to perform the mutational analysis.

In TCR, 47 paraffin-embedded tissue samples were obtained (82.5% of total incident cases) and again two of the samples did not contain sufficient DNA to perform mutational analysis.

Finally, mutational analysis was performed in 108 paraffin-embedded tissue samples, 81.8% of the cohort. We sequenced 297 loci from 3 cancer-related genes (KIT, PDGFR and BRAF only in wild type GIST). All cases were c-KIT positive in immunohistochemistry except two, which were c-KIT negative but CD-34 positive.

Table 2 shows the distribution of mutations according to tumor site, using the International Classification of Diseases for Oncology, third version (ICD-O-3) [26]. We found GIST located in the stomach (C-16), small intestine (C17), colon (C18), rectum (C19) and peritoneum (C48.1). No GIST was found in the esophagus. Tumors registered with C48.0 or C48.2 (retroperitoneum and peritoneum) or C76 (abdomen not otherwise specified) were reviewed and reclassified according to a more specific site. The distribution of mutations according to the risk groups defined by Fletcher et al. [13] is also shown in Table 2.

**Table 2:** **Distribution of c-KIT and PDGFR-α genes mutations (** ***N*** **= 108)**

	**c-KIT exon 11**		**c-KIT exon 9**		**PDGFR-alpha exon 12**		**PDGFR-alpha exon 18**		**Wild type**	
	***N***	**%**	***N***	**%**	***N***	**%**	***N***	**%**	***N***	**%**
Site (ICD-O-3 code)										
Stomach (C16)	31	53.5	2	25	1	50	13	100	15	55.5
Small intestine (C17)	23	39.6	6	75	0		0		11	40.7
Colon (C18)	0		0		0		0		1	3.8
Rectum (C20)	1	1.7	0		0		0		0	
Peritoneum (C48.2)	3	5.2	0		1	50	0		0	
Fletcher’s risk groups										
LR	9	15.5	2	25	0		8	61.5	14	51.9
IR	13	22.4	4	50	1	50	4	30.8	7	25.9
HR	33	6.9	2	25	1	50	1	7.7	5	18.5
Met	3	5.2	0		0		0		1	3.7

Using International Classification of Diseases for Oncology third version (ICD-O-3) and according to Fletcher et al. groups of risk. LR, low risk (includes very low risk); IR, intermediate risk; HR, high risk; Met, diagnosed in metastatic disease.

Fifty-eight GIST had exon 11 c-kit mutation, corresponding to 53.7% of the analyzed cases. Of these, 30 cases (51.7%) were GIST localized in the stomach, 24 (41.4) in the small intestine, 3 (5.2%) in the peritoneum without more specific localization and one (1.7%) in the rectum. The most frequent codons involved in exon 11 c-KIT gene mutations were 557 in 12 cases (20.7%), 558 in 14 cases (24.1%), 559 in 11 cases (18.9%) and 560 in 7 cases (12.1%). Mutations involving both codons 557 and 558 were identified in 9 cases, all diagnosed with a high-risk or metastatic GIST and all except one case had recurred at the last follow-up. Table 3 describes the type of mutation for each c-KIT and PDGFR-α exon.

**Table 3:** **Type of mutation in the 108 GIST cases analyzed**

**Exon mutated**	**Type of mutation**	**Number of cases**
Exon 11 gene c-KIT	V559D	8
	W557_K558del	7
	V560D	4
	L576P	3
	R558_L589insDHKWEFPRN	2
	Y570_P577del	2
	H580_K581insIDPTQLPY	2
	V559A	2
	V555_V559del	2
	V560_E561del	2
	T574_P577del	2
	V557_K561del	1
	W557_E561del	1
	W557_E562del	1
	T574_Q575insDP	1
	V559G	1
	Q556_V559 > H	1
	Q556_T574del	1
	P585S	1
	P577S	1
	N567_Q575del	1
	N567_P577 > Y	1
	N566Y	1
	V560del	1
	K558R	1
	K558_V560del	1
	K558_G565del	1
	K550_P551insK	1
	W557_V559del	1
	E554_K558del	1
	D579del	1
	557-558 del	1
	I563_P577del	1
Exon 9 gene c-KIT	Y503_F504insAY	8
Exon 18 gene PDGFR-α	D842V	11
	D842_H845del	1
	I843_D846del	1
Exon 12 gene PDGFR-α	D591Y	1
	W559_V561del	1
Wild type		27

We obtained eight mutations of c-KIT exon 9 (7.4%), two of them (25%) localized in the stomach and six (75%) in the small intestine. All of them were an insertion type mutation Y503_F504insAY.

Thirteen GIST with mutation in exon 18 of the PDGFR-α gene were identified, corresponding to the 12.1% of the samples analyzed. All were located in the stomach and most of them (53.8%) classified as low-risk GIST. Twelve cases had mutations affecting codon 842 (11 cases D842V substitution-missense mutation, one case D842_H845 deletion) and one was a I843_D846 deletion, which also corresponded to the only aggressive case in patients carrying this type of mutation.

Two cases had mutations of gene PDGFR-alpha exon 12 (1.9%). Twenty-seven cases (25.0%) were wild-type, of those 16 cases (59.3%) were localized in the stomach, 10 (37%) in the small intestine and 1 (3.7%) in the peritoneum.

No BRAF mutations (searched for only in wild type cases), nor any mutations of exon 13 or 17 of the c-KIT gene were found.

Only 4 of those 108 GIST patients for which mutational analysis was performed were diagnosed initially with metastatic disease, 3 of them with mutation in exon 11 of c-KIT gene and 1 wild type GIST. So, GIST with localized disease had in a 60.6% of cases mutations of gene c-KIT (63 patients), 14.4% of cases mutations of gene PDGFR-alpha (15 patients) and 25% (26 cases) of wild type tumors.

We found a significant correlation between mutation type and distribution of patients in risk groups (chi-square test *p* value of 0.03), due to the predominance of high-risk tumors in GIST with the exon 11 c-KIT mutation and the predominance of low-risk GIST in the exon 18 PDGFR-α mutation. We also found differences between mutation type and GIST location (chi-square test *p* value 0.008), as the majority of GIST with mutations in the exon 18 PDGFR-α gene were in the stomach.

### Survival analysis

For the whole cohort (132 cases), 10-year OS and RS were 52.8% (*N* at risk 41; 95% confidence interval (CI): 44.6 to 62.5) and 66.0% (95% CI: 55.8 to 78.0), respectively. Table 4 shows 5- and 10-year survival by risk groups defined by Fletcher et al. [13] and the AFIP [18] classifications. In four cases, we did not find sufficient information to classify. Table 5 shows survival according to mutational status.

**Table 4:** **Five- and 10-year observed and relative survival of GIST according to risk of malignant behavior classifications**

	**Time (years)**	***N*** **at risk**	**OS (95% confidence interval)**	**RS (95% confidence interval)**
All cases (*N* = 132)				
	5	84	62.9 (55.2 to 71.7)	69.7 (61.1 to 79.5)
	10	41	52.8 (44.6 to 62.5)	66.0 (55.8 to 78.0)
Fletcher et al. classification				
LR	5	34	82.5 (71.5 to 95.2)	88.1 (76.4 to 101.6)
	10	19	73.6 (60.7 to 89.3)	88.4 (72.9 to 107.2)
IR	5	22	75.0 (60.6 to 92.9)	89.1 (70.2 to 113.1)
	10	12	71.1 (56.0 to 90.2)	81.8 (66.1 to 101.3)
HR	5	28	56.2 (42.8 to 72.2)	63.0 (49.1 to 80.9)
	10	11	39.1 (26.9 to 57.0)	49.0 (33.6 to 71.3)
Met	5	2	8.3 (1.3 to 54.4)	9.0 (1.4 to 58.6)
AFIP classification				
LR	5	46	83.3 (74.0 to 93.9)	91.5 (81.2 to 103.1)
	10	26	74.4 (63.2 to 87.6)	90.7 (77.1 to 106.8)
IR	5	14	61.9 (44.3 to 86.6)	69.2 (49.5 to 96.8)
	10	7	55.0 (36.6 to 82.7)	70.5 (46.9 to 106.0)
HR	5	24	56.1 (42.8 to 73.5)	62.1 (47.3 to 81.4)
	10	9	38.2 (24.9 to 58.6)	46.1 (30.1 to 70.7)
Met	5	2	8.3 (1.3 to 54.4)	9.0 (1.4 to 58.6)

LR, low risk (includes very low risk); IR, intermediate risk; HR, high risk; Met, diagnosed in metastatic disease.

**Table 5:** **Five- and 10-year observed and relative survival of GIST**

**Type of mutation**	**Time (years)**	***N*** **at risk**	**OS (95% confidence interval)**	**RS (95% confidence interval)**
c-KIT exon 11	5	35	59.6 (48.2 to 73.8)	66.3 (53.6 to 82.1)
	10	15	50.6 (38.5 to 66.5)	65.7 (50.0 to 82.1)
c-KIT exon 9	5	8	87.5 (67.3 to 100.0)	87.5 (67.3 to 100.0)
	10	4	60.0 (33.1 to 100.0)	65.3 (36.1 to 108.8)
PDGFR-α exon 18	5	12	84.6 (67.1 to 100.0)	89.7 (71.1 to 106.0)
	10	7	84.6 (67.1 to 100.0)	108.8 (86.3 to 128.6)
Wild type	5	22	75.0 (60.6 to 92.9)	83.6 (67.5 to 103.5)
	10	14	67.0 (51.3 to 87.4)	79.9 (61.3 to 104.3)

According to type of c-KIT or PDGFR-α gene mutation (*N* = 108).

One of our aims was to correlate survival according to the risk groups defined by Fletcher et al. [13] and mutational status. The small numbers of cases made this impossible for all mutation types except the exon 11 c-KIT gene (Table 6) and exon 18 PDGFR-α gene.

**Table 6:** **Five- and 10-year observed and relative survival of GIST with exon 11 mutation**

**Fletcher group**	**Time (years)**	***N*** **at risk**	**OS (95% confidence interval)**	**RS (95% confidence interval)**
Low and very low	5	7	66.7 (42.0 to 100.0)	73.3 (46.2 to 109.9)
	10	4	66.7 (42.0 to 100.0)	84.3 (53.1 to 126.5)
Intermediate	5	11	76.9 (57.1 to 100.0)	82.8 (61.5 to 107.7)
	10	6	76.9 (57.1 to 100.0)	96.5 (71.7 to 125.5)
High	5	19	56.2 (41.4 to 76.4)	62.2 (45.8 to 84.5)
	10	7	40.0 (24.8 to 64.6)	49.2 (30.5 to 79.5)

(*N* = 58) According to risk of malignant behavior of Fletcher et al. classification.

Patients with mutations in exon 11 of the c-KIT gene had a 5-year OS and RS of 59.6% and 66.3%, respectively.

Patients with mutation in exon 18 PDGFR-α gene had an 85.7% 5-year observed survival (*N* at risk = 7; 95% CI: 63.3 to 100.0) and 89.5% 5-year relative survival (95% CI: 66.1 to 104.4) for low and very low-risk groups and 100% 5-year OS and RS (*N* at risk = 4) for the intermediate group. Only one patient diagnosed with a GIST with mutation in this exon was classified in the high-risk group and progressed and died within 3 years of diagnosis.

Table 7 shows the results of the multivariate analysis for observed survival in which age under 65 years old and low risk tumors compared with metastatic tumors were the two variables that achieved statistical significance. The comparison between high- and low-risk tumors achieves statistical significance when excluding in the model the patients with a second neoplasm (HR = 4.53; 95% CI 1.60 to 12.8; *p* value <0.001). Results did not change when AFIP’s classification was used instead of Fletcher’s classification. In univariate analysis, age and risk classification achieved statistical significance and sex, site and type of mutation did not.

**Table 7:** **Multivariate analysis of risk factors for observed survival**

**Variable**	**HR**	***p*** **value**
Age (>65 vs 0 to 64 years old)	2.22 (1.11 to 4.41)	*0.02*
Sex (men vs women)	1.78 (0.96 to 3.26)	0.06
Type of mutation		
KIT 09 vs KIT 11	1.46 (0.44 to 4.79)	0.53
PDGRF vs KIT 11	1.07 (0.4 to 2.87)	0.90
WT vs KIT 11	1.21 (0.56 to 2.57)	0.63
All other vs KIT 11	0.83 (0.44 to 1.58)	0.58
Fletcher’s risk groups		
IR vs LR	1.58 (0.65 to 3.88)	0.32
HR vs LR	2.27 (0.96 to 5.37)	0.06
Met vs LR	11.49 (2.9 to 45.54)	*<0.001*
Site		
C17 vs C16	1.08 (0.57 to 2.05)	0.82
All other vs C16	1.23 (0.33 to 4.58)	0.76

Data in italics show significance.

HR, hazard ratio; LR, low risk (includes very low risk); IR, intermediate risk; HR, high risk; Met, diagnosed in metastatic disease; C16, stomach; C17, small intestine.

### Second cancer risk assessment

Thirty patients, 22.73% of the total cohort, had a diagnosis of another neoplasm. Of those, eight patients had this neoplasm before GIST and were discarded for the final risk analysis.

Twenty-two tumors were evaluable for second cancer risk. Eleven were synchronic tumors (three colon cancers, two rectal cancers, two lung cancers and one gastric, breast, prostate and endometrium cancer, respectively). Eleven cases were metachronic cancers (two breast and urinary bladder cancers and one lung, esophagus, prostate, colon, biliary tree and pancreatic cancer).

Men with GIST had an increased risk of second cancer with SIR of 2.17 (CI 95% 1.15 to 3.71) and also was in women with SIR of 3.08 (95% CI 1.40 to 5.88). For both sexes together, SIR was of 2.47 (95% CI 1.54 to 3.374).

Of those 22 patients, 14 died due to cancer, but only 2 of them due to GIST.

## Discussion

Our population-based study analyzes the distribution of mutational status and survival across the whole spectrum of GIST.

We found a predominance of c-KIT mutations of exon 11(53.7%) and 7.4% of mutations of exon 9, which correlates with data from other population-based studies published in Norway [27], Italy [28], Switzerland [29], Slovakia [30] and France [31]. By contrast, however, in our series, there is a high percentage (25%) of wild-type tumors which do not normally exceed 10% to 15%. This may be due to the use of old samples of paraffin-embedded tissue, which can result in failure to detect mutations because of tissue fixation defects or degradation of the DNA (21 of the 27 wild-type cases were diagnosed prior to 2003).

One important finding is the distribution of tumors with mutation of exon 18 in the PDGFR-α gene according to the risk groups defined by Fletcher et al. [13]. A great percentage of these, as high as 92.3%, were found in less aggressive GIST: very low, low or intermediate risk. These findings are closer to data published by Rössle et al. [32], who did not find any mutations in exons 12 or 18 in the PDGFR-α gene in a population study for the mutational spectrum of metastatic GIST in Switzerland. Emile et al. [33] and Heinrich et al. [34] found less than 3% of patients with exon 18 PDGFR-α mutation in a population-based and clinical trial in advanced GIST. By contrast, however, in another population-based study, Cassier et al. [31] found this type of mutation to be more frequent than we did in high-risk GIST.

Moreover, 61.2% of cases with mutation of exon 11 of the c-KIT gene in our series were diagnosed in a high-risk group or with metastatic disease, similar to that published by Cassier et al. [31] in a population-based study in France.

In contrast to other series, no exon 13 or 17 of the c-KIT gene mutation was found in our study. Exon 17 mutations account for fewer than 1% of cases in almost all published series [27,28,31] and mutations of exon 13 were described in 3.7% of cases by Cassier et al. [31], 3.3% by Steiger et al. [27] and no cases were found by Braconi et al. from 104 samples [28]. As explained previously, we analyzed BRAF mutation only in wild-type GIST and did not find any mutated cases, in contrast to the 13% published by Hostein et al. [10].

Regarding localization of the primary tumor, those cases with mutation in exon 11 of the c-KIT gene showed the same distribution as for GIST as a whole, that is, almost 60% of neoplasm in the stomach and 40% in the small intestine. What is remarkable in our series is that all cases with mutations of exon 18 in the PDGFR-α gene were gastric GIST and 75% of exon 9 c-KIT mutations were in the small intestine. A predominance of extra-gastric localization and poor prognosis has also been described for this type of mutation [35].

Focusing on survival, our data suggests a good survival for patients with mutation of exon 18 of the PDGFR-α gene, in accordance with the fact that these patients were diagnosed with a less aggressive GIST than those with another type of mutation and predominantly in a gastric location. Despite this, we did not find differences between types of mutation in multivariate analysis. This finding must be taken into account as some trials established less sensitivity to imatinib in GIST with D842V type of mutation in exon 18 PDGFR-α gene [36]. Moreover, some authors had reported lower survival in PDGFR-α mutated GIST in patients with more advanced disease than our series [37].

In addition, the 5- and 10-year observed and relative survival of patients with mutation of exon 11 is slightly, although not significantly, lower than the cohort as a whole, reflecting a predominance of high-risk GIST.

The low incidence of GIST in the area of Tarragona compared with other published studies may mean that some less aggressive GIST have been missed. We included cases diagnosed before the Consensus Workshop of 2001 [12] and after that the improvement in the knowledge of GIST could have raised the diagnosis of those and consequently the incidence [38].

In this population-based analysis, GIST patients have an increased risk of developing a second cancer and most of them are diagnosed synchronously. Recently, Phan et al. [39] published the results of SEER (Surveillance Epidemiology and End Results program) in which the rate of second cancer after GIST was 7.07%. The SIR between observed and expected second neoplasm was 2.03 (CI 0.34 to 2.05) in the pre-imatinib era (1992 to 2001) and 1.27 (CI 0.96 to 1.66) in the imatinib era (2002 to 2009), with an increased risk for colon adenocarcinoma and renal cell carcinoma after GIST in the imatinib era.

It was not possible in our series to perform a statistical analysis of the risk of developing another cancer following GIST by pairs of each second neoplasm, due to the small number of cases, although we found a higher percentage of second tumors than SEER study, reflecting maybe a more exhaustive case-finding procedure. The study of the existence of shared etiologic factors between GIST and other neoplasm deserves further investigation.

## Conclusions

Our population-based study shows a mutation-type distribution in the c-KIT and PDGFR-α genes in GIST patients similar to that found in other population-based studies and a survival of the most frequent mutation, exon 11 c-KIT gene, similar to that of the GIST group as a whole.

GIST with an exon 18 PDGFR-α mutation are, in our population-based analysis, less aggressive and display better survival, although they may be difficult to treat with systemic therapies at recurrence.

In our population-based study, GIST patients have an increased risk of developing a second neoplasm.

## Abbreviations

AFIP: Armed Forces Institute of Pathology
CI: confidence interval
GCR: Girona Cancer Registry
GIST: gastrointestinal stromal tumors
IARC: International Agency for Research in Cancer
ICD-O: International Classification of Diseases for Oncology
OS: observed survival
PCR: polymerase chain reaction
PDGFR-α: platelet-derived growth factor receptor-alpha
RS: relative survival
SIR: standardised incidence ratio
TCR: Tarragona Cancer Registry
